# The Hatoyama Cohort Study: Design and Profile of Participants at Baseline

**DOI:** 10.2188/jea.JE20120015

**Published:** 2012-11-05

**Authors:** Hiroshi Murayama, Mariko Nishi, Yumiko Shimizu, Mi-Ji Kim, Hiroto Yoshida, Hidenori Amano, Yoshinori Fujiwara, Shoji Shinkai

**Affiliations:** 1 Research Team for Social Participation and Community Health, Tokyo Metropolitan Institute of Gerontology, Tokyo, Japan; 1 東京都健康長寿医療センター研究所社会参加と地域保健研究チーム; 2 Research Team for Promoting Independence of the Elderly, Tokyo Metropolitan Institute of Gerontology, Tokyo, Japan; 2 東京都健康長寿医療センター研究所自立促進と介護予防研究チーム

**Keywords:** cohort profile, community-dwelling elderly, frailty, functional decline, Hatoyama Cohort Study

## Abstract

**Background:**

Investigation of frailty among elderly adults and development of prevention strategies to address this are critical in delaying progression of functional decline and thus extending healthy life expectancy. However, there has been no Japanese epidemiologic cohort study of frailty. The Hatoyama Cohort Study was launched in 2010 to identify factors that predict functional decline and to establish strategies to prevent frailty among community-dwelling elderly Japanese. This report describes the study design and the profile of the participants at baseline.

**Methods:**

The Hatoyama Cohort Study is a prospective study of community-dwelling individuals aged 65 years or older living in the town of Hatoyama in Saitama Prefecture, Japan. Comprehensive information, including socioeconomic status, physiological indicators, physical, psychological, and cognitive function, social capital, neighborhood environment, and frailty, was collected in a baseline survey using face-to-face interviews in September 2010. Survival time, long-term care insurance certification, and medical and long-term care costs after the baseline survey will be followed. In addition, a follow-up survey will be conducted in the same manner as the baseline survey every 2 years.

**Results:**

A total of 742 people participated in the baseline survey (mean age: 71.9 ± 5.2 years, men: 57.7%, living alone: 7.7%). Almost all participants were independent in their daily lives, and approximately 10% were categorized as frail on the *kaigo-yobo* (care prevention) checklist.

**Conclusions:**

The Hatoyama Cohort Study is expected to contribute to the development of strategies that prevent frailty in later life and extend healthy life expectancy in Japan’s rapidly aging society.

## Abstract (ja)

**【背景】:**

高齢期の虚弱の解明とその予防策の検討は、生活機能低下の進展を遅らせ、健康余命を延伸させるために重要である。しかし、虚弱に焦点を当てた疫学研究は未だ日本に存在しない。日本人地域在住高齢者の生活機能低下の予測因子を検討し、虚弱予防に向けた対策を確立するため、2010年に鳩山コホート研究を立ち上げた。本報では、鳩山コホート研究の研究デザインとベースライン時における研究参加者の特性について記述する。

**【方法】:**

鳩山コホート研究は、埼玉県鳩山町に在住する65歳以上の高齢者を対象にした前向き研究である。2010年9月、面接方式によるベースライン調査において、社会経済的状態、生理学的・身体的・心理的・認知的機能、ソーシャルキャピタル、近隣環境、そして虚弱指標を含む包括的情報が収集された。死亡、転出、介護保険認定、医療・介護給付費に関する情報をベースライン調査以降追跡している。また、ベースライン調査と同様の形式を用い、2年毎の追跡調査を実施することにしている。

**【結果】:**

ベースライン調査には742名が参加した（平均年齢71.9±5.2歳、男性57.7%、独居7.7%）。ほぼ全員が日常生活動作は自立しており、約10%が「介護予防チェックリスト」によって虚弱と判定された。

**【結論】:**

急速な高齢化が進む日本において、鳩山コホート研究は後期高齢期の虚弱予防、および健康余命の延伸に向けた対策の確立に寄与することが期待できる。

## INTRODUCTION

Extending healthy life expectancy is a major challenge in a rapidly aging society, and aging is more prominent in Japan than in any other nation. It has been estimated that the number of Japanese aged 75 years or older in 2050 (24 million; proportion of population, 26.5%) will be double that in 2005.^1^ The trajectory of functional decline among elderly adults can be classified into 3 types: early onset (20%), minimal decrement (60%), and late onset (13%) (the other 7% represents early deaths).^2^ In Japan, the growing proportion of elderly adults with late-onset functional decline will become increasingly important in the near future.

Frailty is a clinical condition associated with a high risk of functional decline and is highly prevalent in elderly adults,^3^ increasing their vulnerability to adverse health outcomes such as disability, need for long-term care, hospitalization, and mortality.^4^^,^^5^ Exploring the trajectory of frailty and devising strategies to prevent it are of growing importance in attempts to intervene early in disability, slow the progression of functional decline, and, ultimately, extend healthy life expectancy. Evidence indicates that health and functional status in elderly adults cannot be explained by a single factor.^6^^,^^7^ Therefore, it is essential to comprehensively investigate the effects of multiple factors on frailty.

Although a cohort study that examines a variety of factors (eg, physiological, physical, psychological, social, and neighborhood environmental factors) is necessary to address the above questions, to our knowledge, no such study has been conducted in Japan. Thus, we launched the Hatoyama Cohort Study in 2010 to identify factors that predict functional decline and to establish strategies to prevent frailty (including characterizing the trajectory of frailty, identifying predictive factors for frailty, and determining the prognosis for frailty) among community-dwelling elderly Japanese. In this report, we describe the study design and the profile of the participants at baseline.

## METHODS

### Study design, setting, and participants

In this prospective study, the source population comprised community-dwelling individuals aged 65 years or older who lived in the town of Hatoyama in Saitama Prefecture, Japan. Hatoyama is a suburban area located 50 kilometers northwest of central Tokyo and was developed as a commuter town of Tokyo. In June 2010, it had a population of 15 424 (7669 males and 7755 females), with 4028 people aged 65 years or older (proportion, 26.1%). Hatoyama can be divided into an old town and a new town. The old town is a traditional residential and farming area. In contrast, the new town was developed as a new residential area in 1974, at the end of the high-growth period from the mid-1950s through the mid-1970s. Many people from outside Hatoyama moved to live in this new area. As of June 2010, 53.5% of the population lived in the new town area.

To recruit the study participants, we used stratified sampling of 4 groups classified by age (65–74 and 75–84 years) and residential area (old town and new town). People with long-term care certification (level 1–5) and those admitted to hospitals or residing in nursing homes were excluded. The target population for sampling was the 3378 residents in Hatoyama aged 65 to 84 years as of 1 June 2010. For the group of residents aged 65 to 74 years living in the new town, we used a random sampling strategy because the number of target residents was larger than in the other 3 groups. For the other 3 groups, we used a complete census (complete enumeration). The recruitment brochure was mailed to the selected 2697 residents and included detailed information on the study purpose, method, survey items, and merits of study participation. In addition to the random sampling recruitment, we recruited study participants using the Hatoyama town bulletin, to permit broader recruitment. The information included in the town bulletin was identical to that in the recruitment brochure.

After using these 2 methods, 751 people agreed to participate in the Hatoyama Cohort Study (727 by mail-in brochure recruitment; 24 by town bulletin recruitment). There were no monetary or other incentives to encourage participation in this study. Immediately before the baseline survey, we directly informed participants, once again, of the study purpose, method, survey items, and merits of participation, after which 9 declined to participate in the study. As a result, we ultimately included 742 people who agreed to participate in the study (722 recruited by brochure recruitment; 20 by bulletin recruitment). Of 2697 residents recruited by brochure recruitment, the participation rates in the 4 stratified groups were 23.1% (aged 65–74 years in old town), 18.3% (aged 75–84 years in old town), 35.0% (aged 65–74 years in new town), and 27.1% (aged 75–84 years in new town).

### Baseline survey

The baseline survey was administered at the Hatoyama Health Center, located in the town center, during a 6-day period in September 2010. The participants traveled there for the baseline survey, although the survey was conducted at home for 4 participants who were unable to come to the center because of physical impairment or because they were caring for family members.

The Figure shows the conceptual framework of the Hatoyama Cohort Study, which was based on the International Classification of Functioning, Disability and Health.^8^ Variables that might influence frailty and other health outcomes in later life were selected from previous studies^6^^,^^7^^,^^9^ and organized according to this framework. We hypothesized that there is a dynamic interaction among these variables. Table 1 summarizes the main measures surveyed at baseline. All items were measured by physicians, public health nurses, registered nurses, dental hygienists, clinical laboratory technicians, or trained investigators. We stored frozen serum samples for future measurement of inflammatory markers such as C-reactive protein, interleukin-6, and β_2_-microglobulin.

**Figure.**
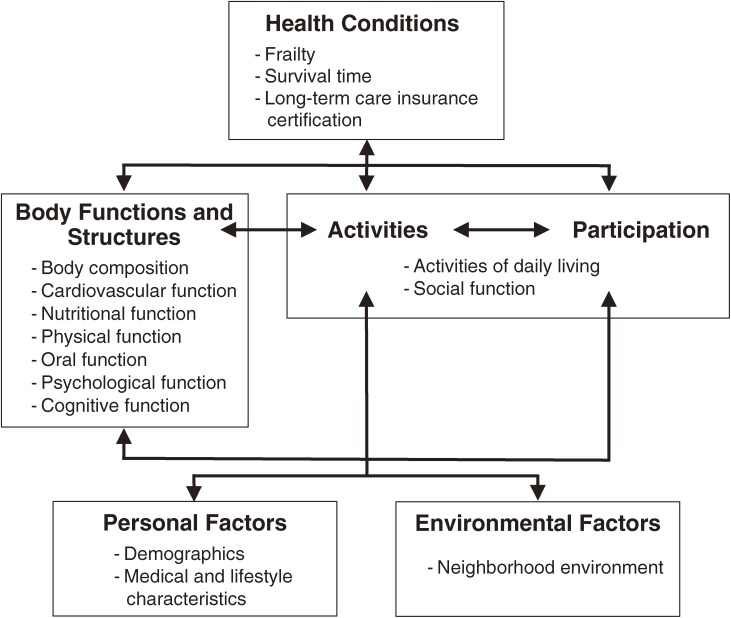
Conceptual framework of the Hatoyama Cohort Study (based on the International Classification of Functioning, Disability and Health).

**Table 1.** Summary of items surveyed in the Hatoyama Cohort Study at baseline, 2010

**Demographics**	
	Living arrangements
	Socioeconomic status
**Medical and lifestyle characteristics**	
	History of physician-diagnosed diseases
	Medication
	Smoking, alcohol consumption, diet, sleeping, physical activity
**Body composition**	
	Body mass index
	Fat mass, fat-free mass, skeletal muscle mass
**Cardiovascular function**	
	Blood pressure and heart rate
	Arterial stiffness (baPWV and ABI)
**Nutritional function**	
	Serum albumin, total cholesterol, blood hemoglobin
	Food intake frequency
**Physical function**	
	Grip strength, walking speed, step length, standing time on 1 foot with eyes open
**Oral function**	
	Chewing and swallowing (RSST)
**Psychological function**	
	Self-rated health
	Depressive mood (GDS short form)
**Cognitive function**	
	Mini-Mental Status Examination
	Trail Making Test Parts A and B
	Delayed word-recall task
**Activities of daily living**	
	Basic activities of daily living
	Higher-order competence of independence (TMIG-IC)
**Social function**	
	Social capital (social network, social support, social participation, general trust, norm of reciprocity)
**Neighborhood environment**	
	Neighborhood living environment (aesthetics, green space, land-use mix)
	Neighborhood social capital (aggregation of individual social capital in the neighborhood)
**Frailty**	
	*Kaigo-yobo* checklist	
	Fried’s frailty indicators	

baPWV: brachial-ankle pulse wave velocity. ABI: ankle brachial index. RSST: Repetitive Saliva Swallowing Test. GDS: Geriatric Depression Scale. TMIG-IC: Tokyo Metropolitan Institute of Gerontology Index of Competence.

*Demographics*: living arrangements and socioeconomic status. Socioeconomic status includes subjective social class, subjective poverty level, educational attainment, and current employment.

*Medical and lifestyle characteristics*: history of physician-diagnosed disease, medication, smoking, alcohol consumption, diet, sleeping, and physical activity.

*Body composition*: body mass index (BMI), fat mass, fat-free mass, and skeletal muscle mass. BMI was calculated from measured height and weight. Fat mass, fat-free mass, and skeletal muscle mass were estimated from bioelectrical impedance analysis by using the InBody 720 (Biospace Inc., Seoul, Korea).

*Cardiovascular function*: blood pressure, heart rate, and arterial stiffness. Arterial stiffness was evaluated by brachial-ankle pulse wave velocity (baPWV)^10^ and the ankle brachial index (ABI).^11^ baPWV and ABI were measured by a volume-plethysmographic device, form PWV/ABI (Omron Colin Co., Ltd., Tokyo, Japan).

*Nutritional function*: serum albumin, total cholesterol, blood hemoglobin, and food intake frequency. Food intake frequency included 10 kinds of foods, eg, meats, vegetables, and fish.^12^

*Physical function*: grip strength, usual and maximum walking speed, step length, and standing time on 1 foot with eyes open.

*Oral function*: chewing and swallowing. Chewing function was assessed using color-changeable chewing gum (Lotte Co., Ltd., Tokyo, Japan), and swallowing function was assessed using the Repetitive Saliva Swallowing Test.^13^^,^^14^

*Psychological function*: self-rated health and depressive mood. The Geriatric Depression Scale, short-form with 15 items was used.^15^^,^^16^

*Cognitive function*: cognitive status was assessed using the Mini-Mental State Examination (MMSE),^17^^,^^18^ the Trail Making Test parts A and B,^19^ and the delayed word-recall task using the Japanese version of the word booklet for the Alzheimer’s Disease Assessment Scale—cognitive component.^20^

*Activities of daily living (ADL)*: basic ADL (BADL) and higher-order competence of independence. BADL was measured using 5 items: walking, eating, bathing, dressing, and toileting. To measure higher-order competence, we used the Tokyo Metropolitan Institute of Gerontology Index of Competence, which consists of 3 subscales: instrumental self-maintenance, intellectual activity, and social role.^21^

*Social function*: social network, social support, social participation, general trust, and norm of reciprocity were evaluated as dimensions of social capital. These items were developed for this study. We asked about the frequency of contact with family/relatives and friends, excepting cohabitating family members, as a measure of the participants’ social network. Social support included availability of instrumental, informational, emotional, and appraisal support from various sources.

*Neighborhood environment*: neighborhood living environment and neighborhood social capital. We asked about respondents’ perception of their local living environment, using several items—including aesthetics, green space, and access—that were developed for this study. Hatoyama consists of 28 small districts (*chou-chou* in Japanese). As neighborhood-level environmental measures, we aggregated individual-level responses on living environment and social capital in every district and calculated the district average score for each item.

*Frailty*: We assessed frailty status by using 2 indices. The first was an index developed as the *kaigo-yobo* (care prevention) checklist to screen for frailty.^22^ This index consists of 15 self-administered items, including the dimensions of being homebound, nutrition, and falls. The index score ranges from 0 to 15, and a higher score indicates a greater likelihood of frailty. The cut-off point was set between 3 and 4, and a score of 4 or higher was classified as frailty. The other frailty index was developed by Fried et al^4^ and is one of the most extensively used in the world. It includes 5 indicators: shrinking, weakness, exhaustion, slowness, and low activity (measured by weight loss, grip strength, 2 items from the Center for Epidemiologic Studies Depression Scale, walking speed, and physical activity, respectively). The score ranges from 0 to 5, and a higher score indicates a greater likelihood of frailty. A score of 3 or higher is classified as frailty, and a score of 1 or 2 is classified as pre-frailty.

### Follow-up outcome surveys

We will follow 4 types of outcomes: health outcomes, survival time, long-term care insurance (LTCI) certification, and medical and long-term care costs. To measure individual changes in health outcomes, we will conduct a follow-up survey every 2 years in the same manner as the baseline survey. To monitor survival time, LTCI certification, and medical and long-term care costs since the baseline survey (on September 2010), every month we will confirm information on date of death or movement out of the study area, LTCI certification status, and medical and long-term care costs, which will be provided by the town of Hatoyama.

### Ethical considerations

The study protocol was reviewed and approved by the Ethical Committee of Tokyo Metropolitan Institute of Gerontology (approved on 5 August 2010). All participants gave written consent to participate in this study.

### Statistical analysis

To describe the characteristics of the study participants at baseline, we compared the main measures among participants grouped by age and sex and by age and residential area using the chi-square test for nominal variables, the Kruskal-Wallis test for ordinal variables, and 1-way analysis of variance for continuous variables. Statistical significance was set as a *P* value less than 0.05 on a 2-tailed test. The statistical analyses were performed using IBM SPSS 19.

## RESULTS

Among the 742 participants, mean age was 71.9 ± 5.2 years, 57.7% were men, and 7.7% were living alone. Almost no participants had a BADL dependency, and more than 90% had contact with others more than once a week and felt that they could receive social support. About 60% of participants had a baPWV greater than 1700 cm per second. Approximately 10% were categorized as frail on the *kaigo-yobo* checklist. There were no important differences in the demographic characteristics of participants recruited by mail-in brochure and those recruited by town bulletin; participants in the latter group were all aged 65 to 74 years (data not shown).

Table 2 shows the characteristics of participants by age and sex. Educational duration of 13 years or longer, long-term white-collar employment, and current smoking were more frequent in men than in women in both age groups. A score of 26 or lower on the MMSE and frailty were more frequent in people aged 75 or older as compared with those aged 65 to 74 in both men and women. Not having a current job and being divorced or widowed were more frequent in women aged 75 or older than in the other groups. The proportion of daily drinking among men aged 75 or older was higher than that among men aged 65 to 74 (45.5% vs 16.9%); no such trend was observed in women.

**Table 2.** Baseline characteristics of the Hatoyama Cohort Study (2010) by age and sex

			All	Men		Women		*P*-value
				
				65–74 y	≥75 y	65–74 y	≥75 y	
Number of individuals			742	305	123	213	101	
**Demographics**								
	Age	mean ± SD	71.9 ± 5.2	69.2 ± 2.7	78.5 ± 2.8	68.7 ± 2.7	79.0 ± 2.8	0.269
	Sex	men, %	57.7	—	—	—	—	
	Family members	living alone, %	7.7	4.3	9.8	9.4	11.9	0.248
		2, %	46.6	52.8	44.7	46.9	29.7	
		≥3, %	46.0	42.9	45.5	43.7	58.4	
	Marital status	married, %	82.8	93.4	85.4	81.6	50.0	<0.001
		divorced/widowed, %	16.2	4.9	13.8	18.0	50.0	
		unmarried, %	0.9	1.6	0.8	0.5	0.0	
	Subjective social class	upper, %	3.6	3.7	1.6	4.7	4.0	0.019
		middle, %	82.7	82.6	77.2	84.9	85.2	
		lower, %	13.6	13.8	21.1	10.3	10.9	
	Subjective poverty level	affluent, %	18.7	18.4	17.0	20.3	18.8	0.374
		middle, %	72.7	73.4	69.1	71.7	77.2	
		poor, %	8.5	8.2	13.9	8.0	4.0	
	Years of education	≥13, %	34.2	50.5	41.5	18.5	9.0	<0.001
	Long-term occupation	blue-collar, %	25.4	26.2	36.6	17.5	25.7	<0.001
		white-collar, %	52.0	73.8	63.4	28.8	20.8	
		unemployed/housewife, %	22.7	0.0	0.0	53.8	53.5	
	Current job	yes, %	22.8	30.8	18.7	19.3	10.9	<0.001
**Medical and lifestyle characteristics**								
	Smoking	current smoking, %	11.5	19.0	13.0	4.2	2.0	<0.001
	Alcohol consumption	every day, %	29.2	16.9	45.5	7.0	3.0	<0.001
		sometimes, %	18.1	21.7	17.9	15.0	13.8	
		none/rarely, %	52.7	31.5	36.6	78.0	83.2	
**Body composition**								
	BMI	<18.5, %	5.3	2.6	7.0	6.7	8.7	0.088
		18.5–25.0, %	64.4	66.7	54.8	69.2	59.8	
		≥25.0, %	30.2	30.8	38.3	24.1	31.5	
**Cardiovascular function**								
	baPWV (cm/s)	<1400, %	7.3	6.9	2.5	12.7	3.0	<0.001
		1400–1700, %	29.5	32.7	16.5	39.2	15.0	
		≥1700, %	63.2	60.4	81.0	48.1	82.0	
	ABI	<0.9, %	1.5	1.7	1.7	0.9	2.0	0.292
		0.9–1.0, %	1.0	0.3	3.3	0.9	0.0	
		≥1.0, %	97.6	98.0	95.0	98.1	98.0	
**Nutritional function**								
	Serum albumin (g/dl)	mean ± SD	4.3 ± 0.3	4.3 ± 0.3	4.2 ± 0.3	4.4 ± 0.3	4.3 ± 0.2	<0.001
	Total cholesterol (mg/dl)	mean ± SD	203 ± 35	196 ± 32	191 ± 36	218 ± 35	205 ± 33	<0.001
	Blood hemoglobin (g/dl)	mean ± SD	13.3 ± 1.3	13.9 ± 1.3	13.5 ± 1.4	12.8 ± 1.0	12.4 ± 1.2	<0.001
**Physical function**								
	Grip strength (kg)	mean ± SD	28.2 ± 8.2	34.8 ± 5.6	30.4 ± 5.5	21.9 ± 4.2	18.6 ± 4.2	<0.001
	Usual walking speed (m/s)	mean ± SD	1.2 ± 0.3	1.2 ± 0.2	1.1 ± 0.3	1.2 ± 0.2	1.0 ± 0.2	<0.001
**Psychological function**								
	Self-rated health	good, %	85.6	88.2	76.4	88.3	83.1	0.008
	GDS (range: 0–15)	≥6, %	12.6	9.8	17.2	12.2	15.8	0.140
**Cognitive function**								
	MMSE (range: 0–30)	≤26, %	11.7	8.6	18.9	7.1	22.2	<0.001
**Activities of daily living**								
	BADL independency		99.9	99.7	100.0	100.0	100.0	0.698
	Instrumental self-maintenance (range: 0–5)	full points, %	96.0	96.4	93.5	98.6	92.1	0.021
	Intellectual activity (range: 0–4)	full points, %	83.0	85.2	78.0	85.8	76.2	0.052
	Social role (range: 0–4)	full points, %	72.7	68.9	67.5	79.7	76.2	0.020
**Social function**								
	Social network	contact with others (except cohabitating family members) more than once a week, %	91.5	87.2	91.1	95.8	96.0	0.002
	Social support	receiving instrumental support, %	99.2	99.0	97.6	100.0	100.0	0.081
		receiving informational support, %	98.5	98.4	95.9	99.5	100.0	0.035
		receiving emotional support, %	98.4	97.7	96.7	99.5	100.0	0.097
		receiving appraisal support, %	95.3	95.1	88.6	98.6	97.0	<0.001
	General trust	yes, %	86.0	87.8	88.6	82.5	85.2	0.293
	Norm of reciprocity	yes, %	84.6	84.6	80.3	87.3	84.2	0.411
**Neighborhood environment**								
	Aesthetics	good aesthetics, %	81.4	81.3	76.4	85.3	80.0	0.224
	Green space	extensive green space, %	90.6	92.2	88.6	89.6	90.0	0.643
	Access	good access, %	44.3	45.3	49.6	40.5	43.0	0.428
**Frailty**								
	*Kaigo-yobo* checklist (range: 0–15)	≥4 (frailty), %	10.6	7.5	18.6	4.0	24.8	<0.001

Missing values were removed.

*P* value for the difference among 4 groups.

BMI: body mass index. baPWV: brachial-ankle pulse wave velocity. ABI: ankle brachial index. GDS: Geriatrics Depression Scale. MMSE: Mini-Mental State Examination. BADL: basic activities of daily living.

BADL independence was defined as independence in all 5 types of BADL.

Table 3 shows the characteristics of participants by age and residential area. There were some differences in socioeconomic status between participants in the old and new areas of the town. As compared with participants in the new town area, greater proportions of respondents in the old town area felt that they were in a lower social class and were less educated, in both age groups. Moreover, participants in the new town were more likely to have long-term white-collar employment than those in the old town in both age groups. The proportion of participants scoring 26 or lower on the MMSE was lower among new-town residents as compared with old-town residents, particularly among participants aged 65 to 74 (4.1% vs 15.3%). Regarding neighborhood environment, new-town residents evaluated their living environment more highly than did those in the old town in both age groups.

**Table 3.** Baseline characteristics of the Hatoyama Cohort Study (2010) by age and residential area

			Old town		New town		*P*-value
				
			65–74 y	≥75 y	65–74 y	≥75 y	
Number of individuals			178	81	340	143	
**Demographics**							
	Age	mean ± SD	69.2 ± 2.7	79.0 ± 2.8	68.9 ± 2.7	78.6 ± 2.8	<0.001
	Sex	men, %	53.4	48.1	61.8	58.7	0.080
	Family members	living alone, %	6.2	7.4	6.5	12.6	<0.001
		2, %	36.0	18.5	57.9	49.0	
		≥3, %	57.8	74.1	35.6	38.4	
	Marital status	married, %	82.5	63.0	91.8	73.2	<0.001
		divorced/widowed, %	14.7	37.0	8.0	26.1	
		unmarried, %	2.8	0.0	0.3	0.7	
	Subjective social class	upper, %	2.8	2.5	4.7	2.8	<0.001
		middle, %	77.5	74.1	86.7	84.7	
		lower, %	19.6	23.5	8.6	12.6	
	Subjective poverty level	affluent, %	10.7	19.8	23.6	16.8	<0.001
		middle, %	76.4	71.6	70.8	73.4	
		poor, %	12.9	8.6	5.6	9.8	
	Years of education	≥13, %	18.1	12.3	47.5	35.2	<0.001
	Long-term occupation	blue-collar, %	41.6	54.3	12.7	18.9	<0.001
		white-collar, %	39.9	27.2	63.4	53.8	
		unemployed/housewife, %	18.5	18.5	23.9	27.3	
	Current job	yes, %	39.3	32.1	19.2	5.6	<0.001
**Medical and lifestyle characteristics**							
	Smoking	current smoking, %	12.4	8.6	13.2	7.7	0.278
	Alcohol consumption	every day, %	28.7	17.3	31.5	31.5	0.006
		sometimes, %	16.3	12.3	20.3	18.2	
		none/rarely, %	55.0	70.3	48.2	50.4	
**Body composition**							
	BMI	<18.5, %	3.0	6.7	5.0	8.3	0.219
		18.5–25.0, %	64.5	61.3	69.5	54.5	
		≥25.0, %	32.5	32.0	25.5	37.1	
**Cardiovascular function**							
	baPWV (cm/s)	<1400, %	8.5	3.8	9.8	2.1	<0.001
		1400–1700, %	34.5	21.3	35.8	12.8	
		≥1700, %	57.1	75.0	54.4	85.1	
	ABI	<0.9, %	0.6	1.3	1.8	2.1	0.205
		0.9–1.0, %	0.0	1.3	0.9	2.1	
		≥1.0, %	99.4	97.5	97.3	95.7	
**Nutritional function**							
	Serum albumin (g/dl)	mean ± SD	4.3 ± 0.3	4.2 ± 0.3	4.4 ± 0.2	4.2 ± 0.2	<0.001
	Total cholesterol (mg/dl)	mean ± SD	198 ± 35	199 ± 40	209 ± 35	196 ± 32	<0.001
	Blood hemoglobin (g/dl)	mean ± SD	13.1 ± 1.3	12.6 ± 1.5	13.6 ± 1.2	13.2 ± 1.3	<0.001
**Physical function**							
	Grip strength (kg)	mean ± SD	28.3 ± 8.3	24.4 ± 7.7	30.1 ± 8.0	25.6 ± 7.6	<0.001
	Usual walking speed (m/s)	mean ± SD	1.2 ± 0.2	1.1 ± 0.3	1.2 ± 0.2	1.1 ± 0.3	<0.001
**Psychological function**							
	Self-rated health	good, %	85.4	85.1	89.8	76.2	0.002
	GDS (range: 0–15)	≥6, %	15.2	13.6	8.5	18.3	0.015
**Cognitive function**							
	MMSE (range: 0–30)	≤26, %	15.3	23.8	4.1	18.4	<0.001
**Activities of daily living**							
	Instrumental self-maintenance (range: 0–5)	full points, %	96.1	90.1	97.9	94.4	0.010
	Intellectual activity (range: 0–4)	full points, %	78.7	66.7	89.1	83.2	<0.001
	Social role (range: 0–4)	full points, %	74.2	76.5	72.9	68.5	0.562
**Social function**							
	Social network	contact with others (except cohabitating family members) more than once a week, %	92.7	96.3	89.7	91.6	0.247
	Social support	receiving instrumental support, %	98.9	100.0	99.7	97.9	0.172
		receiving informational support, %	97.2	98.8	99.7	97.2	0.067
		receiving emotional support, %	97.2	100.0	99.1	97.2	0.149
		receiving appraisal support, %	94.9	95.1	97.3	90.8	0.024
	General trust	yes, %	78.5	82.7	89.4	85.5	0.003
	Norm of reciprocity	yes, %	85.4	80.0	85.8	83.2	0.573
**Neighborhood environment**							
	Aesthetics	good aesthetics, %	76.4	74.1	86.4	80.3	0.009
	Green space	extensive green space, %	84.8	77.7	94.4	95.7	<0.001
	Access	good access, %	20.2	28.4	55.5	57.0	<0.001
**Frailty**							
	*Kaigo-yobo* checklist (range: 0–15)	≥4 (frailty), %	5.8	19.0	6.1	22.6	<0.001

Missing values were removed.

*P* value for the difference among 4 groups.

BMI: body mass index. baPWV: brachial-ankle pulse wave velocity. ABI: ankle brachial index. GDS: Geriatrics Depression Scale. MMSE: Mini-Mental State Examination.

## DISCUSSION

We have described the study design and baseline profile of participants in the Hatoyama Cohort Study, which was launched in 2010. We found notable differences in socioeconomic status and neighborhood characteristics between participants living in the old and new areas of the town regardless of age. These 2 areas have unique characteristics. The new town area has rapidly developed as a new residential area, while the old town remains a traditional residential area with extensive farm land. These attributes might explain the differences in socioeconomic status and neighborhood environment observed among the participants in the 2 areas. It is possible that these differences will have effects on health outcomes such as prevalence of frailty, functional decline, survival time, and LTCI certification.

The strengths of the Hatoyama Cohort Study are as follows. First, we are comprehensively investigating multiple gerontological factors that could influence frailty and functional decline. Several objective measurements are also being evaluated, such as body composition, cardiovascular and physical function, and physiological indicators. Second, face-to-face interviews were used to collect data for the baseline survey and will again be used in follow-up surveys. This method contributes to building a complete database. Third, we will be able to collect data on several types of follow-up outcomes, which are provided by the town of Hatoyama every month. Finally, Hatoyama is a suburb of Tokyo, in which the trend of rapid population aging in Japan will be most prominent.^23^ Findings from this study are expected to assist in developing strategies for healthy aging.

A weakness of the study is that the participation rate was not high. In addition, very few participants had a BADL dependency. Also, about 90% of participants responded that they had a high or moderate income, and about 85% felt their health condition was good. In contrast, results from a Japanese national survey showed that less than 50% of Japanese aged 65 years or older felt they had a high or moderate income, and about 70% felt that their health condition was good.^24^ Thus, it appears that only independent, healthy, and wealthy people became participants, although the mean age and male-female ratio of the participants did not substantially differ from those of the overall population of the town aged 65 to 84 years (mean age, 71.9 ± 5.2; 51.2% men). The possibility of a healthy volunteer effect must be considered.

In conclusion, the Hatoyama Cohort Study was launched in 2010 to identify factors that predict functional decline and to establish prevention strategies for frailty among community-dwelling elderly Japanese. An additional follow-up survey will be conducted every 2 years after the baseline survey. In addition, information on survival time, LTCI certification, and medical and long-term care costs will be collected. The Hatoyama Cohort Study is expected to contribute to extending healthy life expectancy in Japan’s rapidly aging society.

## ONLINE ONLY MATERIALS

The Japanese-language abstract for articles can be accessed by clicking on the tab labeled Supplementary materials at the journal website http://dx.doi.org/10.2188/jea.JE20120015.

Abstract in Japanese.
