# Supplementary material for: The Hatoyama Cohort Study: Design and Profile of Participants at Baseline
Source: J Epidemiol. 2012 Nov 5;22(6):551–8. doi: 10.2188/jea.JE20120015 (PMC3798568; doi:10.2188/jea.JE20120015)
Supplement: Abstract in Japanese. [file je-22-551-s001.pdf]

鳩山コホート研究：研究デザインとベースラインにおける研究参加者の特性

[1]村山洋史、[1]西真理子、[1]清水由美子、[2]金美芝、[1]吉田裕人、[1]天野秀紀、[1]藤原佳典、[1]新開省二
東京都健康長寿医療センター研究所社会参加と地域保健研究チーム[1]
東京都健康長寿医療センター研究所自立促進と介護予防研究チーム[2]

【背景】高齢期の虚弱の解明とその予防策の検討は、生活機能低下の進展を遅らせ、健康余命を延伸させるために重要である。しかし、虚弱に焦点を当てた疫学研究は未だ日本に存在しない。日本人地域在住高齢者の生活機能低下の予測因子を検討し、虚弱予防に向けた対策を確立するため、2010 年に鳩山コホート研究を立ち上げた。本報では、鳩山コホート研究の研究デザインとベースライン時における研究参加者の特性について記述する。【方法】鳩山コホート研究は、埼玉県鳩山町に在住する 65 歳以上の高齢者を対象にした前向き研究である。2010 年 9 月、面接方式によるベースライン調査において、社会経済的状態、生理学的・身体的・心理的・認知的機能、ソーシャルキャピタル、近隣環境、そして虚弱指標を含む包括的情報が収集された。死亡、転出、介護保険認定、医療・介護給付費に関する情報をベースライン調査以降追跡している。また、ベースライン調査と同様の形式を用い、2 年毎の追跡調査を実施することにしている。【結果】ベースライン調査には 742 名が参加した（平均年齢 71.9±5.2 歳、男性 57.7%、独居 7.7%）。ほぼ全員が日常生活動作は自立しており、約 10%が「介護予防チェックリスト」によって虚弱と判定された。【結論】急速な高齢化が進む日本において、鳩山コホート研究は後期高齢期の虚弱予防、および健康余命の延伸に向けた対策の確立に寄与することが期待できる。

キーワード：コホート研究、地域在住高齢者、虚弱、生活機能低下、鳩山コホート研究
